# Metabolic energy decline coupled dysregulation of catecholamine metabolism in physiologically highly active neurons: implications for selective neuronal death in Parkinson’s disease

**DOI:** 10.3389/fnagi.2024.1339295

**Published:** 2024-02-21

**Authors:** Kandatege Wimalasena, Oluwatosin Adetuyi, Maya Eldani

**Affiliations:** Department of Chemistry and Biochemistry, Wichita State University, Wichita, KS, United States

**Keywords:** Parkinson’s disease, Catecholamine metabolism, neuromelanin, alphasynuclein, catecholamine biosynthesis and regulation, neurodegeneration, oxidative stress

## Abstract

Parkinson’s disease (PD) is an age-related irreversible neurodegenerative disease which is characterized as a progressively worsening involuntary movement disorder caused by the loss of dopaminergic (DA) neurons in substantia nigra pars compacta (SNpc). Two main pathophysiological features of PD are the accumulation of inclusion bodies in the affected neurons and the predominant loss of neuromelanin-containing DA neurons in substantia nigra pars compacta (SNpc) and noradrenergic (NE) neurons in locus coeruleus (LC). The inclusion bodies contain misfolded and aggregated α-synuclein (α-Syn) fibrils known as Lewy bodies. The etiology and pathogenic mechanisms of PD are complex, multi-dimensional and associated with a combination of environmental, genetic, and other age-related factors. Although individual factors associated with the pathogenic mechanisms of PD have been widely investigated, an integration of the findings to a unified causative mechanism has not been envisioned. Here we propose an integrated mechanism for the degeneration of DA neurons in SNpc and NE neurons in LC in PD, based on their unique high metabolic activity coupled elevated energy demand, using currently available experimental data. The proposed hypothetical mechanism is primarily based on the unique high metabolic activity coupled elevated energy demand of these neurons. We reason that the high vulnerability of a selective group of DA neurons in SNpc and NE neurons in LC in PD could be due to the cellular energy modulations. Such cellular energy modulations could induce dysregulation of DA and NE metabolism and perturbation of the redox active metal homeostasis (especially copper and iron) in these neurons.

## 1 Introduction

Parkinson’s disease (PD) is an age-related irreversible neurodegenerative disease which is the second most common among all neurodegenerative diseases. PD is largely characterized as a progressively worsening involuntary movement disorder caused by the loss of dopaminergic (DA) neurons in substantia nigra pars compacta (SNpc). Two main pathological hallmarks of PD are the accumulation of inclusion bodies in the cytosol of affected neurons known as Lewy bodies and the predominant loss of neuromelanin-containing DA neurons in substantia nigra pars compacta (SNpc) and noradrenergic (NE) neurons in locus coeruleus (LC) (Zecca et al., 2004, 2006; Zucca et al., 2006; Rommelfanger and Weinshenker, 2007; Vila, 2019). The Lewy bodies contain misfolded and aggregated α-synuclein (α-Syn) fibrils [however, precise role of α-SYN in PD or its regular physiological function is not fully understood, at present; for recent reviews see (Sulzer and Edwards, 2019; Bernal-Conde et al., 2020; Sharma and Burré, 2023)]. Although environmental, genetic, and other age-related causes of PD have been studied for several decades, an integration of the findings to a unified causative mechanism has not been achieved. Most experimental observations to date suggest that the causes of PD are multidimensional and thus, any integrated mechanism must satisfy the key findings in all relevant areas. Here we envision an integrated mechanism for the degeneration of DA neurons in SNpc and NE neurons in LC in PD using available experimental data.

### 1.1 The hypothesis

The proposed hypothetical mechanism is primarily based on the unique high metabolic activity coupled elevated energy demand of these neurons. We reason that the high vulnerability of a selective group of DA neurons in SNpc and NE neurons in LC in PD could be due to the cellular energy modulations induced dysregulation of DA and NE metabolism and perturbation of the redox active metal homeostasis (especially copper and iron) in these neurons as argued below.

## 2 Critical evaluation and discussion of the evidence for the hypothesis

### 2.1 Catecholamine biosynthesis, storage, and recycling

**SCHEME 1:**
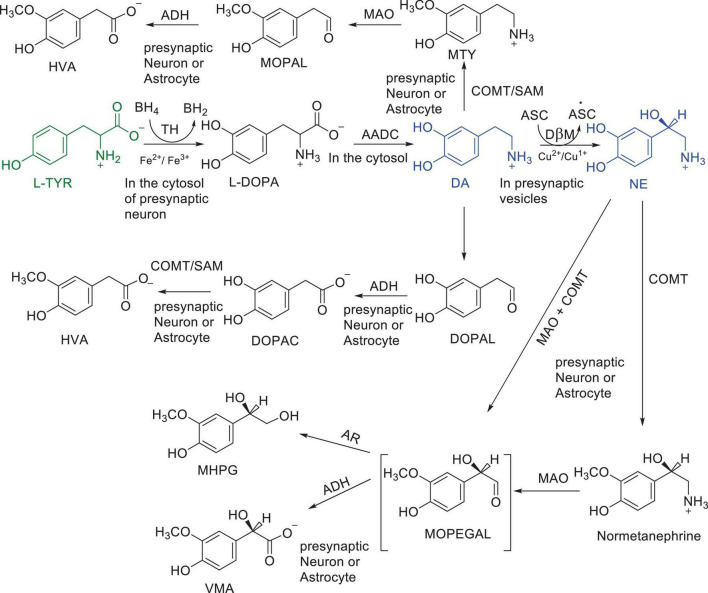
Major pathways of DA and NE metabolism. Enzymes: AADC, aromatic amino acid decarboxylase; ADH, aldehyde dehydrogenase; COMT, catechol O-methyl transferase; DβM, dopamine β-hydroxylase; MAO, monoamine oxidase; TH, tyrosine Hydroxylase Cofactors: ASC, Ascorbic acid; BH4; Tetrahydrobiopterin; SAM S-adenosylmethionine Substrates and products; DA, dopamine; DOPA, L-3,4-dihydroxyphenylalanine; DOPAC, 3,4-dihydroxyphenyacetic acid; DOPAL, 3,4-dihydroxyphenylacetaldehyde; HVA, Homo-vanillic acid; MHPG, 3-methoxy-4-hydroxyphenylglycol; MOPAL, 3-methoxy-4-hydroxyphenyl acetaldehyde; MOPEGAL, 3-methoxy-4-hydroxyphenylglycolaldehyde; MTY, 3-methoxy tyramine; L-NE, L-norepinephrine; L-Tyr, L-tyrosine; VMA, 3-methoxy-4-hydroxymandelic acid.

**SCHEME 2:**
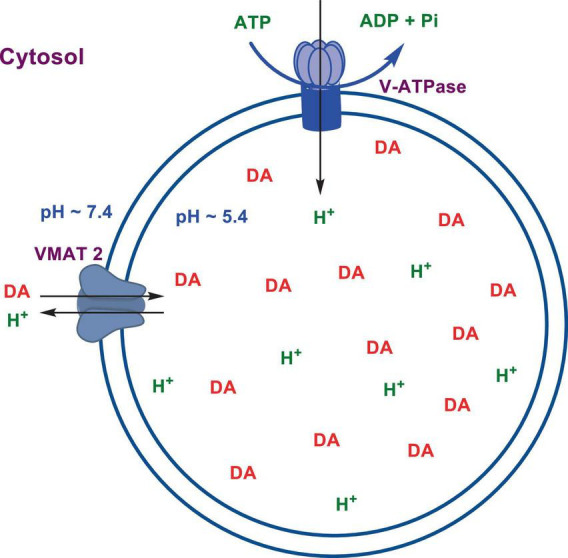
Transient storage of cytosolic DA in dopaminergic synaptic vesicles.

**SCHEME 3:**
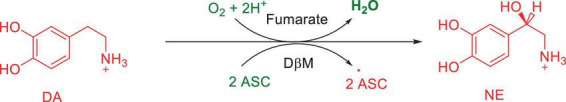
Conversion of DA to NE by dopamine β-monooxygenase (DβM).

**SCHEME 4:**
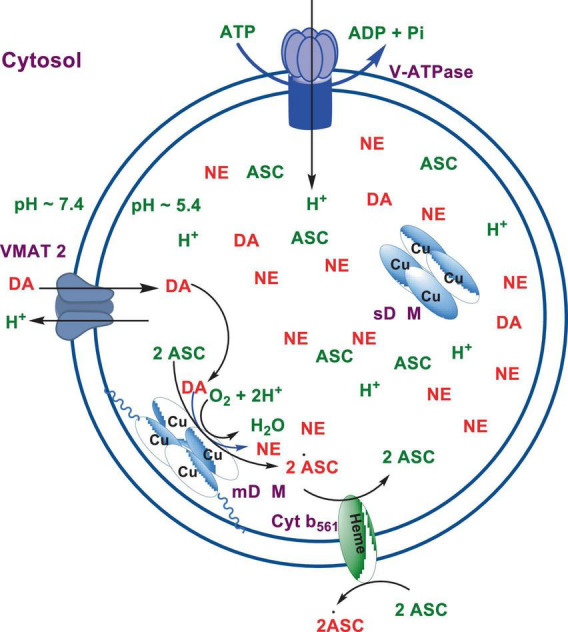
Conversion of DA to NE in synaptic vesicles of adrenergic/noradrenergic neurons.

**SCHEME 5:**
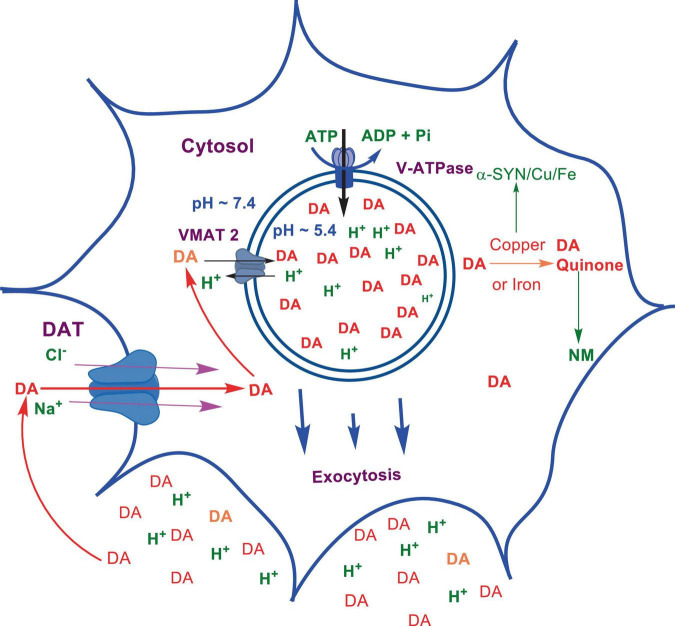
Exocytotic release and recycling of DA in dopaminergic neurons.

**SCHEME 6:**
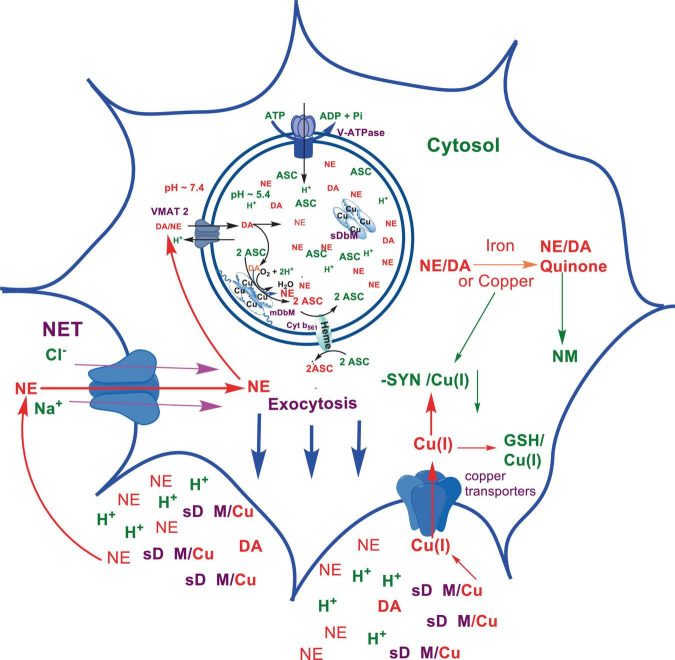
Exocytotic release and recycling of NE in noradrenergic neurons.

Numerous studies suggest that oxidatively labile cytosolic catecholamines, their metabolic intermediates, and biosynthetic precursors such as L-3,4-dihydroxyphenylalanine (L-DOPA) could collectively contribute to the degeneration of SNpc DA and LC NE neurons in PD. The intrinsic oxidative lability of the 3,4-dihydoxy phenyl functionality of DA, NE, their biosynthetic precursors and metabolites make them selectively and significantly more vulnerable to autoxidation under cytosolic conditions (Umek et al., 2018), particularly in the presence of redox active transition metals such as iron or copper, with an elevated tendency to produce increased oxidative stress (Miller et al., 1990). Thus, the perturbations of DA and NE metabolisms leading to increase cytosolic concentrations could cause increased oxidative stress and degeneration of affected neurons. Therefore, it is logical to focus on the biochemical, bioenergetic and biophysiological aspects of biosynthesis, storage, and cellular distributions of DA and NE in their respective neurons, initially [for a general rev. see ref. (Eisenhofer et al., 2004)]. Briefly, the first step in the catecholamine biosynthetic pathway is the conversion of aromatic amino acid, tyrosine, to L-DOPA by iron containing monooxygenase, tyrosine hydroxylase (TH), in the cytosol of catecholaminergic neurons (Scheme 1). TH reaction is the first and rate-limiting step in the catecholamine biosynthetic pathway and is under tight short-term regulation by cytosolic catecholamine mediated feedback inhibition (especially DA) in conjunction with the phosphorylation/dephosphorylation of the regulatory domain Ser residues (40, 31, and 19) by a number of protein kinases/phosphatases (Dickson and Briggs, 2013). In the second step, cytosolic L-DOPA is effectively decarboxylated by a pyridoxal phosphate dependent enzymes, DOPA decarboxylase or non-specific aromatic amino acid decarboxylases (AADC) (Bertoldi, 2014) to DA in the cytosol and, cytosolic DA is actively sequestered into the synaptic vesicles for transient storage through a H^+^ coupled transmembrane antiporter, vesicular monoamine transporter-2 (VMAT2) (Scheme 2; Wimalasena, 2011). The intragranular H^+^ ion gradient required for functioning of VMAT2 mediated active vesicular uptake of DA against a steep concentration gradient is generated by an inward H^+^ translocating V-ATPase in the synaptic vesicle membrane (Wimalasena, 2011). In NE and E neurons, DA is converted to NE by the copper enzyme, dopamine β-monooxygenase (DβM), located inside the synaptic vesicles, employing ascorbic acid as the physiological reductant (Schemes 3, 4; Gonzalez-Lopez and Vrana, 2020). In adrenergic neurons, NE is transported back to the cytosol from the synaptic vesicles and N-methylated by phenyl ethanolamine N-methyltransferase (PNMT) using S-adenosylmethionine (SAM) as a CH_3_ donor (Ziegler et al., 2002) to produce epinephrine (E) in the cytosol and sequestered back into adrenergic synaptic vesicles by a process analogous to DA, employing VMAT2. Furthermore, a fraction of DA and NE released by exocytosis is taken up back into the corresponding presynaptic neurons through Na^+^ and Cl^–^ co-transport coupled plasma membrane DA transporter (DAT) in DA neurons (Scheme 5) or NE transporter (NET) (Torres et al., 2003) in NE neurons (Scheme 6) for recycling. Cytosolic DA or NE derived from re-uptake pathway is combined with newly synthesized cytosolic DA or NE and effectively sequestered into the corresponding synaptic vesicles through VMAT2 as above (Schemes 5, 6).

### 2.2 SNpc DA and LC NE neurons require a large investment of metabolic energy because of their specialized physiological functions in comparison to other DA and NE neurons

The biosynthesis outlined above suggest that catecholaminergic neurons generally require a relatively high investment of metabolic energy for their regulated transmitter biosynthesis, storage, and recycling, in comparison to other types of neurons. For example, energy stored in synaptic vesicle transmembrane pH gradients (Schemes 2, 4) and neuronal plasma membrane Na^+^ and Cl^–^ ion gradients (Schemes 5, 6) are vital for effective synaptic vesicle sequestration and recycling of DA and NE in their respective neurons (Eisenhofer et al., 2004). More importantly, most these processes are interdependent and must be integrated and collectively regulated for optimal functioning of these neurons. Thus, perturbations of one or more of these processes could lead to dysregulation of DA and NE metabolisms leading to harmful downstream effects. For example, our previous studies have shown that the inhibition of V-ATPase or reduction of cytosolic ATP levels dissipate intragranular pH gradients and DA (or NE) gradients within a brief period in resealed chromaffin granule ghosts *in vitro* (Wimalasena et al., 2007). In addition to these general bioenergetic requirements of most DA and NE neurons, unique and specific characteristic of SNpc DA neurons and LC NE neurons require additional metabolic energy for their optimal physiological functions. For example, autonomous pacemaking and L-type Ca^2+^ channel activities (Guzman et al., 2009; Putzier et al., 2009; de Oliveira et al., 2019) specifically associated with these neurons require additional metabolic energy (Ni and Ernst, 2022) for their survival. This is because a continuous influx of extracellular Ca^2+^ into neurons during specialized processes requires efflux back into the extracellular medium through plasma membrane Ca^2+^ ATPase. Alternatively, it can be compartmentalized into endoplasmic reticulum (ER) or mitochondrial Ca^2+^ stores through membrane Ca^2+^ ATPases. These processes occur against steep concentration gradients, ensuring the maintenance of physiological cytosolic Ca^2+^ levels essential for optimal neuronal functioning (Gleichmann and Mattson, 2011; Duda et al., 2016). In addition, extensive axonal arborization with multiple synaptic neurotransmitter release of DA neurons in SNpc and NE neurons in LC require even more metabolic energy to maintain their normal physiological activities (Oertel et al., 2019). Therefore, SNpc DA and LC NE neuronal demand for metabolic energy are even higher than that of regular DA or NE neurons. Thus, an efficient and continuous mitochondrial metabolic energy production is essential for functioning of SNpc DA and LC NE neurons, since energy requirements of these neurons are generally met by the mitochondrial electron transport coupled oxidative phosphorylation (Exner et al., 2012; Hall et al., 2012). *Consequently, reduction of mitochondrial energy production efficiency due to the inhibition of electron transport chain components by environmental toxins, loss of function mutations in various mitochondrial proteins and factors, or age-related general reduction of the mitochondrial metabolic energy production efficiency could be detrimental to the physiological functions of these neurons. Reduced cellular energy supply* (Exner et al., 2012) *could result in the dysregulation of SNpc DA and LC NE metabolisms leading to increased oxidative stress associated downstream adverse effects. Thus, the selective degeneration of SNpc DA and LC NE neurons in PD could be due to a combination of environmental* (Ball et al., 2019), *genetic* (Klein and Westenberger, 2012), *and age-related factors associated with inefficient mitochondrial metabolic energy production* (Bose and Beal, 2016) *leading to dysregulation of DA and NE metabolism in these neurons.*

### 2.3 NM was spontaneously produced from oxidized DA or NE in metabolically highly active SNpc DA and LC NE neurons as a defensive mechanism against the metabolic energy coupled frequent dysregulation of DA and NE metabolism

**SCHEME 7:**
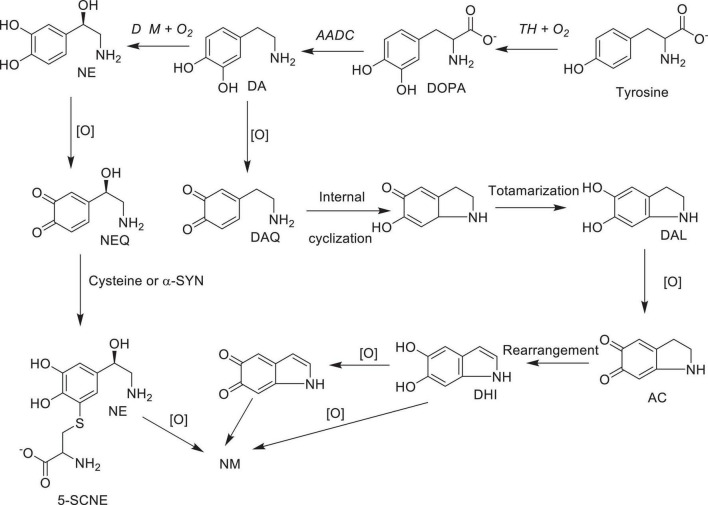
Possible major oxidative pathways for the formation of neuromelanin from dopamine and norepinephrine: AC, aminochrome DA, dopamine; DAL, leuco-dopamine chrome; DAQ, dopamine quinone; DHI, 5,6- hydroxy indole; DOPA L-3,4-dihydroxyphenylalanine; NE, norepinephrine; NEQ norepinephrine quinone; 5SCNE, 5-S-cysteinylnorepinephrine Enzymes: TH, tyrosine hydroxylase; AADC, aromatic amino acid decarboxylase; DβM, dopamine beta-monooxygenase; [O], autoxidation in the presence of transition metal ions; NM, neuromelanin.

As mentioned above, neuromelanin (NM)-containing DA neurons in SNpc and NE neurons in LC are the most vulnerable in PD (Vila et al., 2019). However, the role of NM in PD or the molecular details of NM biosynthesis is not fully understood at present. On the other hand, limited structural information available to date suggest that the initial step of NM biosynthesis is the oxidation of cytosolic DA or NE (but, not synaptic vesicle DA or NE) to the corresponding quinones (Scheme 7; Wakamatsu et al., 2003, 2012, 2019). Although, relatively well understood peripheral melanin biosynthesis is initiated with tyrosinase (or TH) catalyzed oxidation of tyrosine to DOPA and then to dopaquinone (Bisaglia et al., 2007; Nagatsu et al., 2022), since significant levels of tyrosinase expression in SNpc, LC or other areas of the brain has not been detected (Tribl et al., 2007), transition metal (most likely copper or iron) assisted catalytic autoxidation of cytosolic DA or NE to corresponding quinones has been proposed as the initial step in NM production (Sulzer et al., 2000; Zhang et al., 2019). Thus, NM is most likely produced non-enzymatically, from oxidized cytosolic DA or NE and other protein and non-protein components (Sulzer et al., 2000). [Although some derivatives of dopaquinones are neurotoxic (i.e., aminochrome), their toxicity could be mitigated through the reduction by DT-diaphorase which is in turn converted to NM in dopaminergic neurons. In addition, these derivatives stimulate expression of glutathione S-transferase μ 2 (GSTM2) which is then released by astrocytes into inter-synaptic space where it gets internalized by dopaminergic neurons. GSTM2 converts dopaquinone and its derivatives into stable products and eventually to NM and therefore DT-diaphorase and GSTM2 could moderate the neurotoxicity of dopaquinone and its derivatives (Scheme 7 and for a recent review see Segura-Aguilar et al., 2022)]. Interestingly, insoluble NM particles are also known to bind redox active metals including iron and copper (Zecca et al., 2001) and cationic mitochondrial toxins such as MPP^+^ and Paraquat (Capucciati et al., 2021).

*According to the above envisioned high metabolic activity coupled elevated energy demand hypothesis, NM production from the oxidized cytosolic DA or NE could be defined as a defensive mechanism against the low metabolic energy coupled frequent dysregulation of DA and NE metabolism in SNpc DA and LC NE neurons. Metabolically highly active SNpc DA and LC NE neurons with a high demand for metabolic energy* (Ni and Ernst, 2022), *could be significantly more susceptible to frequent low metabolic energy coupled dysregulation of DA and NE metabolism resulting in high levels of NM production*. *On the other hand, frequent DA and NE metabolism dysregulations could also lead to cytosolic DA and NE accumulations, making these neurons more susceptible to increased oxidative stress and eventual degeneration. This occurs when the net rate of cytosolic DA and NE accumulations (biosynthesis plus recycling) exceed the rates of NM production plus VMAT2 mediated synaptic sequestration. Even if these rates were similar, and no net accumulation of cytosolic DA or NE occurs, progressive and prolong cytosolic accumulation of insoluble NM complexes above a certain threshold could interfere with the regular physiological functions of these neurons (e.g., exocytotic release of transmitters and free movement of intracellular organelles such as mitochondria and synaptic vesicles), leading to their malfunctions and degeneration. This proposal is also consistent with the recent demonstration* (Carballo-Carbajal et al., 2019) *that over-expression of human tyrosinase in rat SNpc DA neurons result in age dependent progressive accumulation of NM and their degeneration above specific threshold of accumulation similar to that of PD. Thus*, while the production of neuromelanin (NM) from cytosolic dopamine (DA) or norepinephrine (NE) may initially offer neuroprotection, persistent dysregulation of DA or NE metabolism due to frequent low metabolic energy could result in the accumulation of cytosolic DA or NE. This accumulation, especially beyond a critical threshold, may lead to progressive and selective death of neuromelanin-containing DA and NE neurons in PD. *Thus, metabolically highly active, NM containing SNpc DA and LC NE neurons could be selectively more vulnerable to degeneration in PD. Furthermore, the ability of NM to sequester redox active metal and mitochondrial toxins from the soluble cytosolic milieu will help to maintain the cellular copper and iron homeostasis and to protect electron transport complexes in these neurons form environmental toxins*. *However, under degenerative conditions, NM containing neurons could release intracellular NM complexes containing redox metals to the extra neuronal space stimulating the progression of neural death to the adjacent neurons and activation of microglia induced inflammation* (Liddelow et al., 2017; Tansey et al., 2022), *further stimulating the neural death pathways.*

### 2.4 The physiological role of α-SYN could be to assist the maintenance of cellular toxic metal homeostasis

The early observation that misfolded and aggregated α-SYN containing inclusion bodies (Lewy bodies) are present in SNpc DA neurons of most PD patients has led to the proposal that the accumulation of α-SYN containing Lewy bodies contribute to the dopaminergic degeneration in PD (Lücking and Brice, 2000; Mehra et al., 2019; Srinivasan et al., 2021). *However, whether α-SYN containing Lewy body accumulation is the cause, or it is a downstream symptom of PD pathology has not been convincingly established yet and many questions remain unanswered at present.* For example, total α-SYN levels were consistently reduced in PD patient brains compared to non-PD controls (Stefanis, 2012). Similarly, PD therapies and diagnostic tools developed based on targeting spread, production, aggregation, and degradation of α-SYN have provided a limited or variable success in curing, halting, or diagnosing the progression of PD (Brundin and Olsson, 2011). On the other hand, the ability of α-SYN to bind numerous redox active metals, especially in the context of PD, has been widely studied (Binolfi et al., 2006; Mezzaroba et al., 2019). Among many metals known to bind α-SYN, copper (Wang et al., 2010; Valensin et al., 2016) and iron (Peng et al., 2010) has taken center stage due to the high affinities of these metals toward α-SYN and the general consensus is that these metals are closely associated with the pathophysiology of PD (Montes et al., 2014). The high affinity binding site of α-SYN binds both Cu(I) and Cu(II) with dissociation constants in the nanomolar range [K_*d*_ ∼ 1.10 × 10^–10^ M for Cu(II) (Dudzik et al., 2013) and 3.9 × 10^–9^ M for Cu(I)] (Gentile et al., 2018). Although Fe(II) binds weakly (K_*d*_ = 1.7 × 10^–4^ M), Fe (III) binds strongly (K_*d*_ = 8.3 × 10^–14^ M) to α-SYN (Peng et al., 2010). More importantly, both copper and iron binding to α-SYN increases its tendency to aggregation and formation of insoluble amyloid fibrils *in vitro* (Wang et al., 2010). Additionally, in contrast to the previous proposals that α-SYN bound copper is more effective in redox oxygen activation in the presence of biological reductant such as ascorbate or catecholamines (Valensin et al., 2016), recent studies suggest that binding to α-SYN, reduces oxygen activation efficacy of free copper (Dell’Acqua et al., 2015; Bacchella et al., 2023) further validating the significance of copper binding characteristics of α-SYN. *Thus, it is conceivable that α-SYN could be a physiologically relevant auxiliary in vivo copper/iron buffering system that could contribute to the maintenance of cellular copper/iron homeostasis in SNpc DA and LC NE neurons, especially under stressed conditions.* For example, copper/iron α-SYN interaction could effectively complement other neuronal copper buffering systems such as glutathione [K_*d*_ = 9 × 10^–12^ M (Banci et al., 2010)] and metallothionein, and ferrous binding systems such as ferritin [K_*d*_ = 4.7 × 10^–9^ M (Hulet et al., 1999)]. *Based on these arguments, we propose that*α-synuclein (α-SYN) in dopamine (DA) and norepinephrine (NE) neurons may play a physiological role by serving as an auxiliary cytosolic copper/iron detoxifying/buffering system. These functions protect DA and NE neurons from heightened oxidative stress induced by free copper and iron, particularly in situations where copper/iron homeostasis and/or catecholamine metabolism are dysregulated. *However, as discussed below copper-α-SYN interaction could be physiologically more relevant to the protection of LC NE neurons (in comparison to SNpc DA neurons) from copper/NE induced oxidative stress and downstream adverse effects.*

### 2.5 Copper binding properties of α-SYN could be especially critical to maintain copper homeostasis in LC NE neurons

As mentioned above, the copper enzyme, DβM, catalyzes the conversion of DA to NE in NE and E synaptic vesicles [for reviews see refs (Stewart and Klinman, 1988; Gonzalez-Lopez and Vrana, 2020; Schemes 4, 5)]. Early estimates have demonstrated that equal amounts of sequentially and structurally related (Stewart and Klinman, 1988; Gonzalez-Lopez and Vrana, 2020) membrane bound (mDβM) and soluble (sDβM) forms of DβM are present in NE and E synaptic vesicles (Lewis and Asnani, 1992). After exocytotic release, while sDβM is discarded (Scheme 6), mDβM is recycled through synaptic membrane recycling pathway (Wimalasena and Wimalasena, 2004). The observation that free copper concentration in the synaptic cleft area of NE neurons is relatively higher in comparison to other aminergic neurons under normal physiological conditions (Rihel, 2018) suggest that disposed sDβM may contribute to the increase of free copper in synaptic cleft areas of these neurons. Thus, exocytotic release of the synaptic content of NE neurons must be accompanied by a decrease in intracellular copper levels. Thus, the synaptic release must be coupled with a suitable copper re-uptake system to maintain the overall copper homeostasis in NE neurons. A major portion of extracellular copper enters into neurons through a highly abundant copper transporter 1 (CTR1) (Davies et al., 2013; An et al., 2022) as Cu(I) (An et al., 2022; Scheme 6). Since cytosolic free copper is toxic, especially in the presence of catecholamines, it must be immediately and transiently recruited by medium-affinity intracellular copper buffering systems such as glutathione (K_*d*_ = 9 × 10^–12^ M) or metallothionines (Scheme 6). Subsequently, it is selectively transferred to high affinity copper chaperones (i.e., Atox1, Cox 1, and CCS) in the cytosol (Hatori and Lutsenko, 2016) and eventually recruited for the synthesis of various copper containing proteins. Since metabolically highly active LC NE neurons (see above) must be highly susceptible to frequent dysregulation of cellular copper homeostasis, the copper binding tendency of α-SYN could provide an additional avenue to maintain the neuronal copper homeostasis reducing their vulnerability to produce cytosolic copper/catecholamine induced high oxidative stress. In fact, copper binding and buffering characteristics of α-SYN could be even more critical in PD, since PD pathology is intimately associated with high oxidative stress and significant depletion of cellular reduced glutathione levels (Sian et al., 1994; Martin and Teismann, 2009).

## 3 Conclusion

Salient features of the above envisioned integrated causes of PD are: (1) The high metabolic activity associated elevated energy demand of DA neurons in SNpc and NE neurons in LC makes them selectively more vulnerable to inadequate energy supply associated dysregulation of catecholamine metabolism, increased oxidative stress, and degeneration. (2) Frequent inadequate metabolic energy supply for these neurons could be a consequence of low mitochondrial metabolic energy output, most likely due to the continuous exposure to environmental mitochondrial toxins, age related decline of overall mitochondrial energy production efficiency, or specific mutations in vital mitochondrial proteins and other factors affecting the normal physiological functions of the mitochondria. (3) The selective NM production in PD sensitive DA and NE neurons could be an initial defense mechanism against the inadequate metabolic energy supply coupled dysregulation of catecholamine metabolism. This is because spontaneous insoluble NM production transiently removes toxic cytosolic DA/NE and redox active iron/copper from the soluble cytosolic medium leading to their detoxification. However, frequent dysregulation of DA/NE metabolism leading to their cytosolic accumulations or increased synthesis of insoluble NM complexes above a certain threshold obstructing the normal functions of these neurons could lead to their malfunctions and degenerations. (4) Major physiological function of α-SYN in metabolically highly active SNpc DA and LC NE neurons could be to transiently buffer the redox active cytosolic transition metals (especially, copper and iron). Metal binding stimulated aggregation of insoluble α-SYN/copper/iron complexes facilitate maintenance of the copper and iron homeostasis and lessen the tendency for cytosolic copper/iron/catecholamine induced increase oxidative stress in these neurons. However, progressive, and excessive accumulation of insoluble α-SYN/iron/copper complexes above a specific threshold may interfere with the normal physiological functions of these neurons leading to their malfunctions and degeneration. Taken together, integrated causes envisioned here suggest that long-term preventive strategies of PD must include an adequate continuous supply of cellular metabolic energy to these neurons to minimize the frequent dysregulation of DA and NE metabolisms.

## Data availability statement

The original contributions presented in this study are included in this article/supplementary materials, further inquiries can be directed to the corresponding author.

## Author contributions

KW: Conceptualization, Writing–original draft, Writing−review and editing. OA: Writing–review and editing, Writing–original draft. ME: Writing–review and editing.
